# Association between two CHRNA3 variants and susceptibility of lung cancer: a meta-analysis

**DOI:** 10.1038/srep20149

**Published:** 2016-02-01

**Authors:** Xiao Qu, Kai Wang, Wei Dong, Hongchang Shen, Ying Wang, Qi Liu, Jiajun Du

**Affiliations:** 1 Institute of Oncology, Shandong Provincial Hospital Affiliated to Shandong University, Shandong University, 324 Jingwu Road, Jinan, 250021 P.R. China; 2 Department of Thoracic Surgery, Shandong Provincial Hospital Affiliated to Shandong University, Shandong University, 324 Jingwu Road, Jinan, 250021 P.R. China; 3 Department of Oncology, Shandong Provincial Hospital Affiliated to Shandong University, Shandong University, 324 Jingwu Road, Jinan, 250021 P.R. China

## Abstract

Genome-wide association studies (GWAS) have identified two CHRNA3 polymorphisms (rs578776 and rs938682) associated with lung cancer risk. Furthermore, these polymorphisms were investigated and genotyped by PCR analysis. All eligible case-control studies published up to Mar 1st 2015 were identified by searching Pubmed and Embase database. Negative association between rs578776-T allele and risk of lung cancer was obtained without obvious heterogeneity (OR: 0.83, 95% CI: 0.79–0.86; p = 0.898 for Q test). Rs938682-C allele carriers had a 12% to 28% decreased risk. Genotype model analysis showed results of dominant model for rs578776 (OR with 95% CI: 0.839(0.718–0.981)), dominant model for rs938682 (OR with 95% CI: 0.778(0.663–0.912)) and homozygous model for rs938682 (OR with 95% CI: 0.767(0.708–0.831)) were statistically significant. Subgroup analysis indicated rs578776-T variant had protective effect in Smokers, Caucasians, two histology subgroups, and two match subgroups. Meanwhile, rs938682-C allele was associated with decreased risk in Smokers, Caucasians, Lung cancer, and two match subgroups. Meta-regression suggested ethnicity might be the major source of heterogeneity in allele model and homozygous model for rs938682. Moreover, smoking status might contribute to part of heterogeneity under allele model. In summary, this meta-analysis suggested both rs578776 and rs938682 were significantly associated with the susceptibility of lung cancer.

Approximately, there are estimated 1.3 billion smokers all over the world^1^. Epidemiological evidence indicates that tobacco smoking can exert its pathogenic effect on almost every organ through direct or indirect tobacco exposure, and the smoking associated mortality still remains at a high level for decades^2^. Tobacco smoking, as one of well-known cancer risk factors, was highlighted in the past decades especially for its effect on lung cancer^3^. More than 60 carcinogens could be detected in the tobacco smoke, and they contributed to the procedure throughout from nicotine addiction to lung cancer^4^. Nicotine, as a functional components in the tobacco smoke, could promote tumor cells proliferation, metastasis and inhibit apoptosis through binding to nicotinic acetylcholine receptors (nAChRs) and β-adrenergic receptors (β-ARs), but not initiate tumor genesis^5^. What’s more, the nicotine derivatives 4-(methyl nitrosamino)-1-(3-pyridyl)-1-butanone (NNK) and N-nitrosonor nicotine (NNN) in the tobacco smoke can also bind to nAChRs and strongly induce carcinogenesis^5^. That indicates the genes encoding nAChRs may be associated with susceptibility of lung cancer.

In 2008, three Genome-wide association studies (GWAS) revealed that CHRNA5-A3 cluster situated on chromosome 15q24-25.1 might be potential loci relevant to both nicotine dependence and smoking related cancer^6^,^7^,^8^. From then on, numerous studies have suggested CHRNA3 gene polymorphisms are associated with many types of cancers detection and treatment including lung cancer, gastric cancer, esophageal cancer^9^,^10^,^11^, etc. Other meta-analysis indicated rs1051730^12^, rs6495309^13^, rs12914385, rs8042374^14^ in CHRNA3 were associated with lung cancer risk and part of them even had racial difference. Saccone^15^ proposed the evidence that rs578776 (C > T) could reduce risk of lung cancer in his meta-analysis (OR: 0.82; p value: 9.74E-10) involving 5 databases before 2010. Thereafter, there were several researches in the relation between rs578776 and susceptibility of lung cancer. So far, there has been no meta-analysis focusing the association between rs938682 and risk of lung cancer.

However, the association between both CHRNA3 single nucleotide polymorphisms (SNP, rs578776, rs938682) and susceptibility of lung cancer remains inconsistent. To improve statistical power, we conducted the meta-analysis based on case-control studies to assess the effect of two SNPs on the susceptibility of lung cancer.

## Materials and Methods

### Search strategy

**Figure 1:**
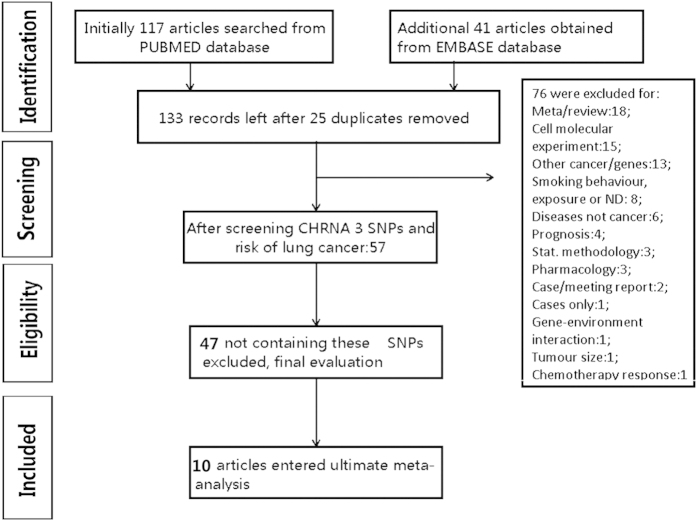
The PRISMA processing map. 10 articles were finally included in the meta-analysis.

PUBMED and EMBASE database were searched by two co-authors separately before March 1^st^ 2015 using combinations of following terms: (CHRNA3 OR CHRNA5-3 cluster OR CHRNA3-CHRNA5-CHRNB4 cluster OR rs578776 OR rs938682 OR 15q25) AND (lung carcinoma OR lung cancer OR lung neoplasm) AND (allele OR genotype OR gene OR polymorphism OR mutation OR variant). The title and abstract of each potential paper was reviewed by two co-authors independently and any irrelevant one was excluded. Process map for the searching details was presented in Fig. 1 under the Preferred Reporting Items for Systematic Reviews and Meta-Analyses (PRISMA) statement^16^.

### Inclusion and exclusion criteria

Articles which met the following criteria were included in this meta-analysis: 1) case-control studies focusing on the association between rs578776, rs938682 and susceptibility of lung cancer; 2) providing the odds risk (OR) and its 95% confidence interval (95% CI) of allele or genotype, or sufficient information to calculate them; 3) Human study and Full-length articles in English. Reasons for exclusion were: 1) no sufficient data; 2) same cohort or duplicated case/control groups; 3) twin or family based studies.

### Data extraction

Data was extracted by two co-authors separately and any difference was dissolved by discussion participated in by more than two authors. Information extracted were: last name of the first author; published year; histology; study design; case and control match; country and ethnicity; smoking status; sample size, gender and age information; frequency, OR and 95% CI of allele and genotype. Data was not presented in primary publication was marked ‘not applicable (NA)’.

### Quality assessment

We assessed the quality of included studies by using a quality score called ‘Extended-Quality Score’ to limit the bias in the meta-analysis. As the primary designers classify the studies into ‘high, median, or poor’ quality, the quality of our included studies was assessed all above 5 points score. The details of this quality assessment version were previous described by Li, *et al.*^17^.

### Statistical analysis

Odds risk (OR) with associated 95% confidence interval (CI) was used to assess the association strength. The pooled OR were calculated for estimating the outcome of five genetic models: Allele model, heterozygous model, homozygous mode, dominant model and recessive model. Z test was applied to test the significance of pooled OR and value of p less than 0.05 was considered as significant. Between-study heterogeneity was calculated through Cochran’s chi-square-based Q statistic test and assessed by value of inconsistency index (I^2^ ranging from 0 to 100%)^18^ whose high value was considered as high heterogeneity. 25%, 50% and 75% were assigned to define low, moderate and high heterogeneity^18^. Pooled ORs were calculated according to DerSimonian-Laird Method which took the between-study variation into the calculation^19^,^20^. Funnel plot was conducted to estimate the potential publication bias. Obvious asymmetric plot reflects publication bias. Egger’s linear regression test was conducted to assess the funnel plot asymmetry and its intercept was determined by the t-test. A less than 0.05 p value of Egger’s test suggested the existence of potential publication bias. Sensitivity analysis and subgroup analysis were feasible methods to find out potential origin of heterogeneity. Subgroups included smoking status, ethnicity, histology and match subgroups. Histology referred to NSCLC and lung cancer (including NSCLC and other pathology subtypes) subgroups. What’s more, we also conducted another method Galbraith plot to test the heterogeneity as a supplement. And points representing researches exceed upper and lower confidence interval regression lines may be potential sources of heterogeneity. Meta-regression was applied to detect the potential heterogeneity among studies. All p values were two sides and less than 0.05 were considered significant. STATA software (Stata Corp, Texas, USA, version 12.0 for Windows) was used to perform this meta-analysis.

## Results

Details of the screening procedure was shown in Fig. 1. After the duplicate removal, the initial 158 articles reduced to 133. The top three excluding reasons were other SNPs (n = 47); meta-analysis and review (n = 18); cell molecular experiment (n = 15). Finally 10 eligible articles in different cases/control groups met the inclusion criteria to evaluate the association two SNPs rs578776, rs938682 in CHRNA3 gene with susceptibility of lung cancer^21^,^22^,^23^,^24^,^25^,^26^,^27^,^28^,^29^,^30^. Additionally, we included the data from Saccone’s article^15^ for there were no published articles reported the impact of rs578776 on lung cancer from these databases except Tseng’s research. Data of Tseng’s research came from a part of EAGLE database^30^. For the polymorphism rs578776, 6 researches and 5 databases were included with total 11763 cases and 12574 controls (Caucasian 11115 cases and 11763 controls). For the polymorphism rs938682, 6 articles involved a total of 6552 cases and 8085 controls (Caucasian cases/controls: 5824/7267; Chinese: 728/818). Genotype for control groups in all articles was informed in the Hardy-Weinberg Equilibrium (p > 0.05). Basic characteristics of all studies were provided in Table 1.

### Impact of rs578776 on the susceptibility of lung cancer

**Figure 2:**
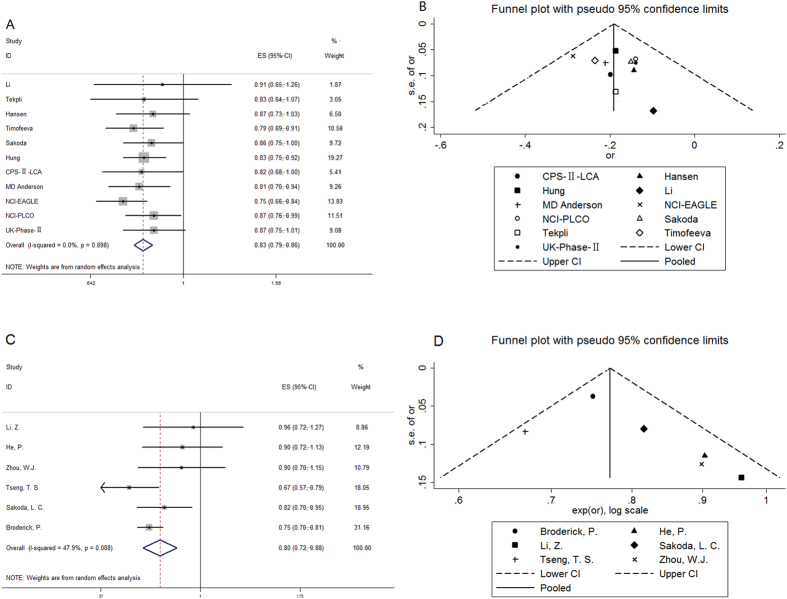
Forest plots of the association between each mutant allele and susceptibility of lung cancer. For each SNP, the mutant allele could decrease (rs578776 and rs938682) the risk of lung cancer without high heterogeneity (Fig. 2A, OR: 0.83, 95% CI: 0.79–0.86, I^2^ = 0.0% for rs578776; Fig. 2C, OR: 0.80, 95% CI: 0.72–0.88, I^2^ = 47.9% for rs938682). Funnel plot for publication bias of two SNPs (rs578776, rs938682). Figure 2B,D showed all researches seemed no obvious asymmetry and had no significant publication bias. P values of Egger’s test for these two SNP were 0.334 and 0.167 respectively.

Overall comparison of rs578776-T allele yielded a remarkably protective effect for lung cancer (pooled OR: 0.83; 95% CI: 0.79–0.86) compared with rs578776-C allele. Meanwhile, there were no evidence of between-study heterogeneity (I^2^ = 0%, p = 0.898, Fig. 2).

Of all studies, the genotype information was available in Li, Tekpli and Sakoda’s articles (Table 2). The included three articles for genotype analysis combined 1256 lung cancer cases and 2025 controls. Significantly reduced lung cancer risk was shown under TT + CT versus CC model (OR: 0.839, 95% CI: 0.718–0.981, p = 0.028) with absent between-study heterogeneity (I^2^ = 0.0%). However, no significant association were detected in the other three models (CC + CT versus TT model, p = 0.189; Homozygous model TT versus CC, p = 0.109; Heterozygous model CT versus CC, p = 0.066) without obvious between-study heterogeneity (I^2^ of all three models 0.0%).

### Impact of rs938682 on the susceptibility of lung cancer

For the polymorphism rs938682-C allele, our meta-analysis gave a pooled OR of 0.80 (95% CI: 0.72–0.88; Z = 4.68, p < 0.001). Moreover, there were evidence of moderate between-study heterogeneity (I^2^ = 47.9%, p = 0.088) under random effect model (Fig. 2).

Four case-control studies included (Li, Skoda, He and Broderick) yielded a total of 5664 lung cancer cases and 6438 controls for genotype analysis. However, Broderick reported pooled OR with 95% CI under homozygous and heterozygous model, and only pooled OR under dominant and recessive model. Thus both heterozygous and homozygous model analysis included Broderick’s research but neither dominant nor recessive model could. Significant decreased risks were observed under both dominant model CC + CT versus TT (pooled OR with 95% CI: 0.778(0.663–0.912), p = 0.002, I^2^ = 0.0%) and heterozygous model CT versus TT (0.767(0.708–0.831), p < 0.01, I^2^ = 0.0%) without statistical heterogeneity. However, the pooled OR was of edge significance with moderate heterogeneity (OR with 95% CI: 0.724(0.520–1.007), p = 0.055, I^2^ = 60.8%) under homozygous model CC versus TT. However, there were no evidence of the association polymorphism rs928682 with the susceptibility of lung cancer (OR with 95% CI: 0.989(0.756–1.296), p = 0.938, I^2^ = 0.0%) under recessive model TT + CT versus CC.

Thus for the analysis of heterogeneity of pooled OR of allele model C versus T and homozygous model CC versus TT, we conducted the Galbraith plot (Supplementary Fig. 2) to test the heterogeneity again, furthermore sensitivity analysis (Supplementary Fig. 1) and subgroup analysis (Table 3) to find out the potential source. Through Galbraith plot, we found all researches might not have significant heterogeneity under allelic model and homozygous model. After deleting a single involving study each time, the alteration of pooled OR and heterogeneity indicated the potential origin of heterogeneity. Through the sensitivity analysis, we found the Tseng’s research and Broderick’s research might be the potential heterogeneity resource of allele and homozygous model respectively. After removing Tseng’s research, we found the pooled OR under allele model remained significant (OR: 0.79, 95% CI: 0.74–0.84, p < 0.001, I^2^: 36.7; p = 0.176). However, I^2^ under homozygous model fell to 0.0% accompanied with p value of pooled OR 0.311. It was worth noting that the sample size of Broderick was much larger than the sum of the other studies (cases: 4417 versus 1247; controls: 4443 versus 1995). That needs to add further relevant studies to verify homozygous model.

### Subgroup analysis and meta-regression

**Figure 3:**
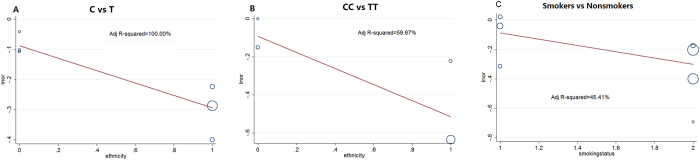
Meta-regression analysis. Impact of ethnicity on the rs938682-C allele heterogeneity was shown in Fig. 3A. 100% between-study variance could be explained by ethnicity. Impact of ethnicity on the rs938682-CC genotype heterogeneity was shown in Fig. 3B. 59.67% variance could be explained by ethnicity. Impact of smoking status on the rs938682-C allele smoking subgroups was shown in Fig. 3C. 45.41% variance could be explained by the smoking status.

To seek for the potential sources of heterogeneity and evaluate the impact of polymorphisms on specific subgroups, we performed subgroup analysis on smoking status, ethnicity, match and histology (Table 3). Rs578776-T allele could significantly reduce the risk of susceptibility of lung cancer in smokers, Caucasians, NSCLC patients, lung cancer patients, match and match not mentioned subgroups. Meanwhile, ethnicity subgroup analysis showed that the protective effect of rs938682-C allele on Chinese might be not as significant as Caucasians (OR: 0.91, 95% CI: 0.79–1.06 for Chinese; OR: 0.75, 95% CI: 0.69–0.81 for Caucasians). And for the smoking status subgroup analysis, risk of smokers with allele C occurring lung cancer was lower than ones with allele T (OR: 0.74; 95% CI: 0.63–0.86). For the Chinese nonsmokers, allele C had no statistically significant effect on susceptibility of lung cancer (OR: 0.92, 95% CI: 0.75–1.13). Moreover, the ORs of rs938682-C allele were significantly attenuated in lung cancer, match, and match not mentioned (NA) subgroups. To evaluate the impact of ethnicity on the heterogeneity of rs938682 allele model and homozygous model, meta-regression was performed (Fig. 3). Proportions of between-study variance explained by ethnicity under allele and homozygous model were 100% and 59.67% respectively. To evaluate the impact of smoking status on between-study heterogeneity, we did the meta-regression on all smoking subgroups. Meta-regression showed that smoking status contributed to 45.41% heterogeneity (Fig. 3).

### Publication bias

Publication bias analysis was shown in Fig. 2 in the form of funnel plot. No significant publication bias was seen in any funnel plot. All researches included were within the pseudo 95% CI limits in funnel plot of three SNPs and showed no significantly asymmetrical. Egger’s test to provide statistical verification of publication bias indicated no evidence of publication bias (p = 0.334 for rs578776 rs578776-T allele model and p = 0.167 for rs938682-C allele model).

### Impact of rs8040868 on the susceptibility of lung cancer

Additionally when screening the published GWAS researches including CHRNA3 gene, we found another independent locus rs8040868 (C > T) might be related to risk of lung cancer. The searching strategy was the same to strategy reported in method part (processing map not shown). Ultimately 1983 cases and 2235 controls for testing rs8040848 were included in 5 articles^22^,^29^,^31^,^32^,^33^. The basic information was shown in Table 1. The rs8040868-T variant might be related to higher susceptibility of lung cancer (OR: 1.17, 95% CI: 1.02–1.33, Z: 2.26, p = 0.024). I^2^ reflected the existence of moderate heterogeneity (I^2^ = 49.6%) and p value was 0.094. Forest plot and funnel plot were shown in supplementary Fig. 3. Funnel plot of the rs8040868 genetic model seemed symmetrical and cooperation with Egger’s test indicated no publication bias (p = 0.783 for rs8040868-T allele model).

## Discussion

To our knowledge, this is the first meta-analysis evaluating the association between rs938682 and lung cancer susceptibility. As a whole, we found these SNPs (rs578776, rs938682) could reduce the risk of lung cancer (pooled OR with 95% CI: 0.83(0.79–0.86) for 578776; 0.80(0.72–0.88) for rs938682). Although potential sources of heterogeneity between researches can’t be easily evaluated or eliminated, we could assume ethnicity and smoking status might be sources through the subgroup analysis especially for rs938682. For Chinese, the association between rs938682 and susceptibility may not be significant (OR: 0.91; 95% CI: 0.79–1.06; p = 0.228). However, the remarkable significance can be detected in the results of GWAS from Caucasians (OR: 0.75; 95% CI: 0.69–0.81; p < 0.001). Through meta-regression, we evaluated the contribution of ethnicity to heterogeneity. Meta-regression indicated that ethnicity contributed to 100% heterogeneity of rs938682 allele model and 59.67% heterogeneity of rs938682 homozygous model. Meanwhile we observe that difference in the association of SNP rs938682 with lung cancer risk may exist in not only ethnicity but also smoking status subgroups. We calculated the total HR and 95% CI in smokers and nonsmokers, and found significance in smokers (HR: 0.74; 95% CI: 0.63–0.86; p < 0.001) and no significance in nonsmokers (HR: 0.92; 95% CI: 0.75–1.13; p = 0.417). However, the nonsmokers were all Asians. To analyze the effect of smoking status and ethnicity on heterogeneity separately, we further did the meta-regression. For overall studies, ethnicity contributed to 100% heterogeneity. For all smoking status subgroups, smoking status contributed to part of heterogeneity (45.41%). That indicated that both ethnicity and smoking status influence the association rs938682-C allele with susceptibility of lung cancer. Additionally when performing the subgroup analysis of histology, we found that heterogeneity within Adenocarcinoma group as a possible result of ethnicity (I-square = 70.9%, p = 0.032 for Q test, data not shown).

After identifying the impact of chromosome 15q25.1 and CHRNA3 gene on the susceptibility of lung cancer by three GWAS in 2008^6^,^7^,^8^, further GWAS and meta-analysis revealed many CHRNA3 polymorphisms relevant to lung cancer. However, rare meta-analysis focused on protective variants. Apart from the risk SNPs, protective variants possess the same importance for cancer screening. What’s more, the mechanism of the association rs578776 and rs938682 with susceptibility of lung cancer was well developed through researches *in Vivo* and *Vitro*.

CHRNA3 encoding α3 nAChR submit was reported related to cell apoptosis and depleting or restoring its expression could induce or resist cell apoptosis^34^. And CHRNA3 may be of vital importance to small cell lung cancer (SCLC) cell viability and play a direct role in lung cancer susceptibility^35^. CHRNA3 polymorphism may contribute to the cancer occurrence through two feasible mechanisms: direct or nicotine dependent cancer promotion. Tseng, *et al.*^30^ reported rs578776 and rs938682 had both direct and total effect on lung adenocarcinoma. What’s more, he drew a conclusion through mediation analysis that there were correlations among nicotine dependence, lung adenocarcinoma and SNPs.

For the reason that nAChRs play a vital role in nervous system and nicotine conduction pathway, recent researches revealed that polymorphisms rs578776 and rs938682 were relevant to neuropsychiatric disease, nicotine addiction and lung cancer^36^,^37^,^38^. Thus the association two polymorphisms with lung cancer might result from direct genetic susceptibility and indirect smoking carcinogenesis. Recent researches put emphasis on the association between rs578776 and smoking behavior in different countries and ethnic groups^39^,^40^. A meta-analysis indicated that rs578776 was relevant to age of first regular tobacco use^41^. rs578776 may also play a vital role in the heaviness of smoking and the age of smoking onset^41^.

However, through the research in rs12914385 and rs8042374 neither rs938682 nor rs578776, Wang, *et al.* emphasized that CHRNA3 might impact indirectly on lung cancer rather than direct effect mainly due to two arguments listed below: variants carriers in CHRNA3 SNPs increase CPD (cigarettes per day); for never smokers the association between SNPs and lung cancer susceptibility may not be significant^42^. To better explore the effect of nicotine exposure, Timofeeva, *et al.*^28^ evaluated the effect of smoking exposure and CHRNA3 gene polymorphisms on the lung cancer occurrence through not only smoking amount (CPD and duration) but also smoking metabolite cotinine level. And he found rs578776 mutant variant T had protective effect for lung adenocarcinoma and the minor allele accompanied with decreased cotinine levels. Moreover, this protective effect might not be significant for the never smokers. However, Tyndale reported the variant allele in rs578776 was irrelevant to CPD but relevant to a lower cotinine level^43^. Moreover, through altering the dACC–thalamus circuit which was reported sensitive to the “status” of smoking rs578776 may influence nicotine dependence through intrinsic reward sensitivity deficit for the smokers^44^,^45^. Thus rs578776 may contribute to the nicotine dependence. What’s more, Wang, *et al.*^46^ proposed some new explanation for the relation between SNPs and lung cancer susceptibility: alteration of nAChRs subunits function and variability in mRNA expression. He reported that rs578776 and rs938682 influenced CHRNA5 mRNA expression. And the level of CHRNA5 mRNA was associated with risk of nicotine dependence. Thus the effect of these two SNPs on the lung cancer risk may partially play a part through this indirect mechanism. Moreover, rs578776 in another research was related to multiple primary cancers even in never smokers^47^. By the subgroup analysis, the rs578776 and rs938682 are both significant in smokers (HR: 0.82 (0.78–0.87) for rs578776; HR: 0.74 (0.63–0.86) for rs938682).

Although not the emphasis in this meta-analysis, rs8040868 allele T mutation was associated with an increased risk of lung cancer (OR: 1.17; 95% CI: 1.02–1.33). The mutation in rs8040868 is a synonymous mutation (do not change the encoding amino acids and the reading frame)^48^; nevertheless, it was reported related to the DNA methylation of CHRNB4 and down regulation of CHRNB4 resulted in reduced proliferation and colonies formation^49^.

In summary, these two SNPs (rs578776 and rs938682) may reduce the risk of lung cancer due to the difference in central nervous system function and nicotine dependence; nicotine metabolism and exposure; effect on other genes expression and function; and direct effect on lung cancer.

However, there are some limitations in our meta-analysis. Firstly, heterogeneity may be the most important problem for the distinct baseline information of cases and controls; study design and implement; lab technique; statistic processing; paper writing and so on. We spared no effort to minimize the heterogeneity by selecting the included paper critically by inclusion and exclusion criteria; extracting and analyzing data strictly by two different authors. Secondly, concrete data of three SNPs can’t be obtained from all GWAS and replication studies. Details needed to perform subgroup analysis can’t be obtained from all articles. We took full advantage of tables, figures and supplementary information to directly extract or indirectly calculate data we need. Thirdly, the amount of researches is not enough to sufficiently evaluate the effect of genotype, smoking behavior, gender, age, histology, ethnicity, and study design on the association between these three SNPs and susceptibility of lung cancer. Lastly, all information was only available in published data. So publication bias, selection bias, or any other potential bias may be inevitable.

In summary, rs578776 and rs938682 are related to susceptibility of lung cancer, and variants in these two SNPs play a protective role. Rs938682-C allele may be significant in Chinese nonsmokers. And rs578776-T allele and rs938682-C allele may be effective for smokers. This research may contribute to screening high risk groups in healthy people and have vital significance for Public Health. Moreover, this may predict further onset of lung cancer in family with cancer history. More researches may be needed to confirm the association between these SNPs with susceptibility of lung cancer and answer new questions whether these SNPs were related to prognosis and response to chemotherapy.

## Additional Information

**How to cite this article**: Qu, X. *et al.* Association between two CHRNA3 variants and susceptibility of lung cancer: a meta-analysis. *Sci. Rep.* **6**, 20149; doi: 10.1038/srep20149 (2016).

## Supplementary Material

Supplementary Information

## Figures and Tables

**Table 1:** basic information extracted from each study.

Author	Year		Histology		Design		Match		Country & ethnicity		Smoking status	
Published Articles												
Li	2012		NSCLC		Hospital		Not mentioned		China, Asian		Nonsmokers only	
Tekpli	2013		NSCLC		Hospital		pack-years		Norway, Caucasian		Nonsmokers/Smokers	
Hansen	2010		lung cancer		population		age, gender and race		America, African		Nonsmokers/Smokers	
Tseng	2014		ADC		population		age, gender		Italy, Caucasian		smokers only	
Timofeeva	2011		lung cancer		population		center, gender, etc		Europe, Caucasian		Nonsmokers/Smokers	
Sakoda	2011		lung cancer		population		age, sex, race, etc		non-Hispanic, Caucasian		smokers only	
Hung	2008		lung cancer		population		sex, age, etc		Europe, Caucasian		Nonsmokers/Smokers	
He	2014		ADC		Hospital		gender and age		China, Asian		Nonsmokers/Smokers	
Zhou	2015		lung cancer		Hospital		Not mentioned		China, Asian		Nonsmokers/Smokers	
Broderick	2009		lung cancer		population		Not mentioned		U.K, Caucasian		Cigarette per day	
Chikova	2012		NSCLC		population		ethnicity		Canada, African		Not analyse	
Amos	2010		lung cancer		Hospital		age, sex, ethnicity		America, African		Nonsmokers/Smokers	
Lou	2014		NSCLC		Hospital		age		China, Asian		Nonsmokers/Smokers	
Data from Saccone’s article												
CPS-II-LCA	2010		lung cancer		population		age, gender, etc		America, Caucasian		smokers	
MD Anderson	2010		NSCLC		Hospital		age, gener, race, etc		America, Caucasian		smokers	
NCI-EAGLE	2010		lung cancer		population		Not mentioned		Italy, Caucasian		smokers	
NCI-PLCO	2010		lung cancer		population		Not mentioned		America, Caucasian		smokers	
UK-Phase-II	2010		lung cancer		population		Not mentioned		U.K, Caucasian		smokers	
Author	Number		Males percent		Mean age		rs578776 T		rs938682 C		rs8040868 C	
	case	control	case	control	case	control	case	control	case	control	case	control
Published Articles												
Li	200	200	67.5	62	57.64	56.66	75.6	77.4	41.2	42.2		
Tekpli	310	348	68.7	77.6	66	60	22.5	26.1				
Hansen	448	611	45.8	45.7	64.1	64.6	49.4	53			34.4	32.5
Tseng	661	1347	79.7	84.9	H	H	27	NA	23	NA		
Timofeeva	894	1805	62.2	61.9	H	H	27	NA				
Sakoda	746	1477	67.3	66.6	H	H	24.5	27.2	19.3	22.6		
Hung	1989	2625	NA	NA	NA	NA	NA	NA				
He	301	318	49	49	59.6	56.1			43	46		
Zhou	228	301	78.1	62.5	58.7	50.2			41.2	45.6	34.2	37.4
Broderick	4417	4443	NA	NA	NA	NA			NA	NA		
Chikova	340	435	59	49	70	40					48	39
Amos	467	388	54.8	41.2	62.4	55.7					39	32.2
Lou	500	500	70	51.8	NA	NA					35.9	35
Data from Saccone’s article												
CPS-II-LCA	699	748										
MD Anderson	1154	1137										
NCI-EAGLE	1770	1340										
NCI-PLCO	1253	1350										
UK-Phase-II	2300	933										

H: hierarchy; ADC: adenocarcinoma; NSCLC: non-small cell lung cancer; NA: Not applicable.

**Table 2:** Pooled OR and 95% CI of genotype analysis.

SNP	Genotype	Studies	OR(95% CI)	Z	p value	I^2^(%)
rs578776	TT + CTvsCC	3	0.839(0.718–0.981)	2.20	0.028	0.0
	CC + CTvsTT	3	1.190(0.918–1.542)	1.31	0.189	0.0
	TTvsCC	3	0.768(0.557–1.061)	1.60	0.109	0.0
	CTvsCC	3	0.858(0.729–1.010)	1.84	0.066	0.0
rs938682	CC + CTvsTT	3*	0.778(0.663–0.912)	3.10	0.002	0.0
	TT + CTvsCC	3*	0.989(0.756–1.296)	0.08	0.938	0.0
	CCvsTT	4	0.724(0.520–1.007)	1.92	0.055	60.80
	CTvsTT	4	0.767(0.708–0.831)	6.54	<0.001	0.0

^*^Broderick’s research didn’t report 95% CI of dominant model and recessive model, only report value of OR.

**Table 3:** Subgroups analysis of allelic model for rs578776 and rs938682.

Subgroups	rs578776				rs938682			
	Studies	OR 95% CI	I^2^(%)	p I^2^	Studies	OR 95% CI	I^2^(%)	p I^2^
Smoking								
smokers	8	0.82(0.78–0.87)	1.4	0.419	4	0.74(0.63–0.86)	36.9	0.191
nonsmokers	2	0.98(0.77–1.24)	0.0	0.494	3	0.92(0.75–1.13)	0.0	0.495
Ethnicity								
East Asian	1	0.91(0.65–1.26)			3	0.91(0.79–1.06)	0.0	0.926
Caucasian	9	0.82(0.78–0.86)	0.0	0.833	3	0.75(0.69–0.81)	32.3	0.228
African	1	0.87(0.73–1.03)						
Histology								
NSCLC	3	0.83(0.73–0.93)	0.0	0.827	3	0.82(0.65–1.04)	71.3	0.031
Mixed	8	0.83(0.79–0.87)	0.0	0.719	3	0.78(0.72–0.85)	20.9	0.282
Matched								
Yes	7	0.83(0.78–0.88)	0.0	0.983	3	0.78(0.66–0.92)	61.3	0.076
NM	4	0.83(0.76–0.90)	21.6	0.281	3	0.83(0.70–0.97)	54.0	0.114

OR: odds ratio; CI: confidence interval; NSCLC: non-small cell lung cancer; NM: not mentioned.
