# Supplementary material for: Association between two CHRNA3 variants and susceptibility of lung cancer: a meta-analysis
Source: Sci Rep. 2016 Feb 1;6:20149. doi: 10.1038/srep20149 (PMC4735583; doi:10.1038/srep20149)
Supplement: Supplementary Information [file srep20149-s1.pdf]

Association between two CHRNA3 variants and susceptibility of lung cancer: a meta-analysis

Running head：Two CHRNA3 polymorphisms were associated with lung cancer.

Xiao Qu[1],*, MD, Kai Wang[1],*, MD, Wei Dong[2], MD，Hongchang Shen[3], MD, Ying Wang[1], MD，Qi Liu[1], §, MD, Jiajun Du[1, 2], §, MD, PHD

[1]Institute of Oncology, Shandong Provincial Hospital Affiliated to Shandong University, Shandong University, 324 Jingwu Road, Jinan, 250021 P.R. China

[2] Department of Thoracic Surgery, Shandong Provincial Hospital Affiliated to Shandong University, Shandong University, 324 Jingwu Road, Jinan, 250021 P.R. China

[3]Department of Oncology, Shandong Provincial Hospital Affiliated to Shandong University, Shandong University, 324 Jingwu Road, Jinan, 250021 P.R. China

* These authors contributed equally to this work.

§Corresponding authors:

Jiajun Du, Department of Thoracic surgery, Shandong Provincial Hospital Affiliated to Shandong University, Shandong University, 324 Jingwu Road, Jinan, 250021 P.R. China; Tel: +86-531-8518-7837; Fax: +86-531-8518-7100; E-mail: dujiajun@sdu.edu.cn

Qi Liu, Institute of Oncology, Shandong Provincial Hospital Affiliated to Shandong University, Shandong University, 324 Jingwu Road, Jinan, 250021 P.R. China; Tel: +86-531-8518-7837; Fax: +86-531-8518-7100; E-mail: liuqi66@sdu.edu.cn.

Supplementary information 1: Sensitivity analysis allele model and homologous model of rs938682

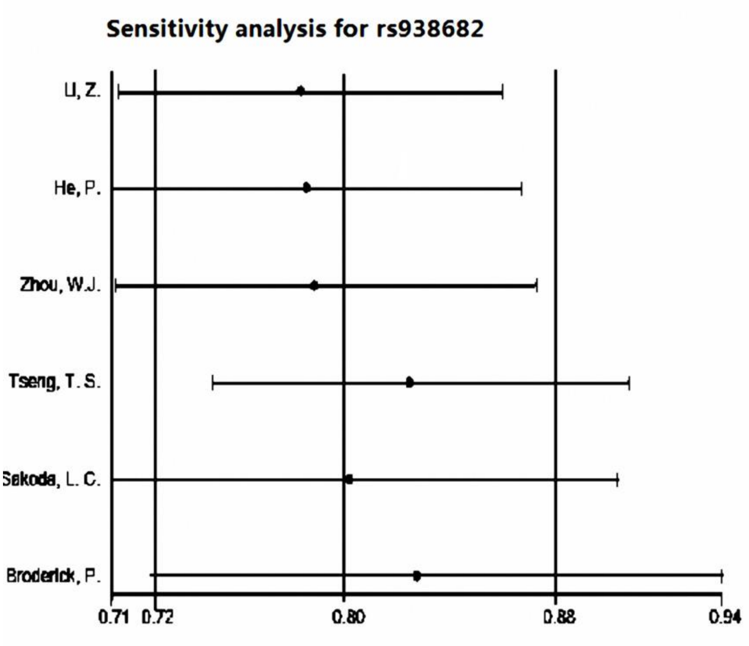

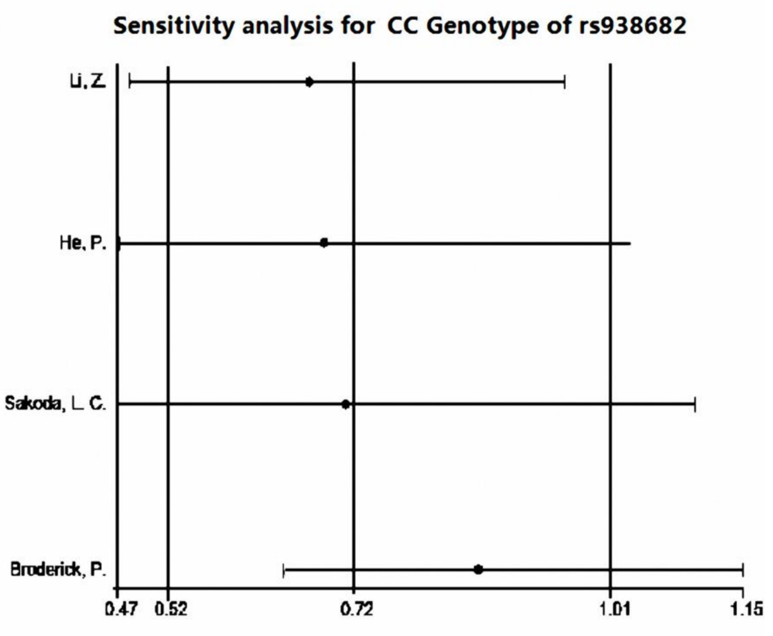

Supplementary information 2: Galbraith analysis allele model and homologous model of rs938682

Galbraith for rs938682 C allele

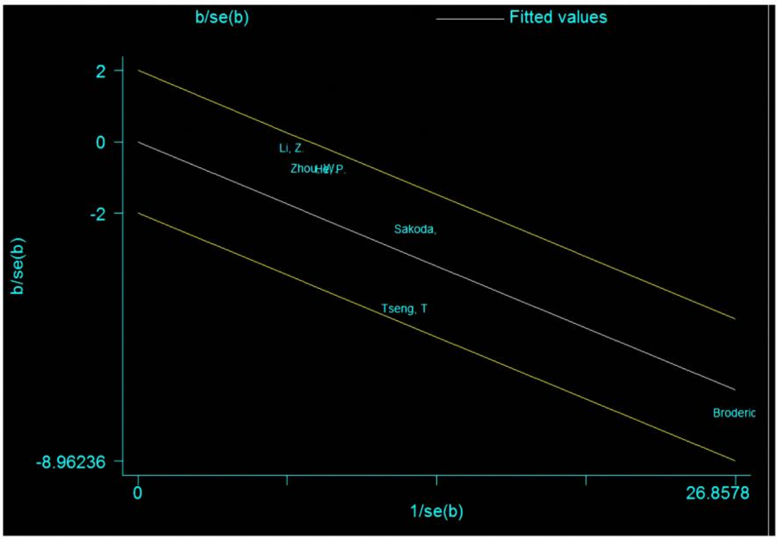

Galbraith for rs938682 CC genotype

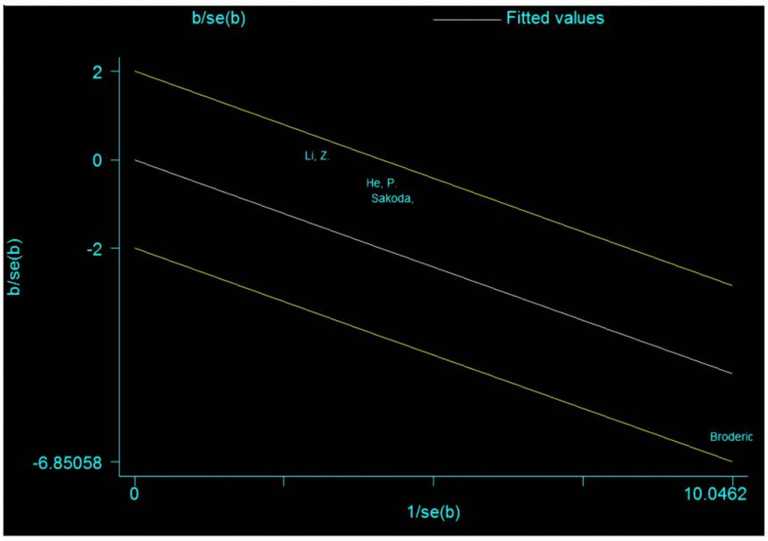

Supplementary information 3: forest plot and funnel plot for rs8040868

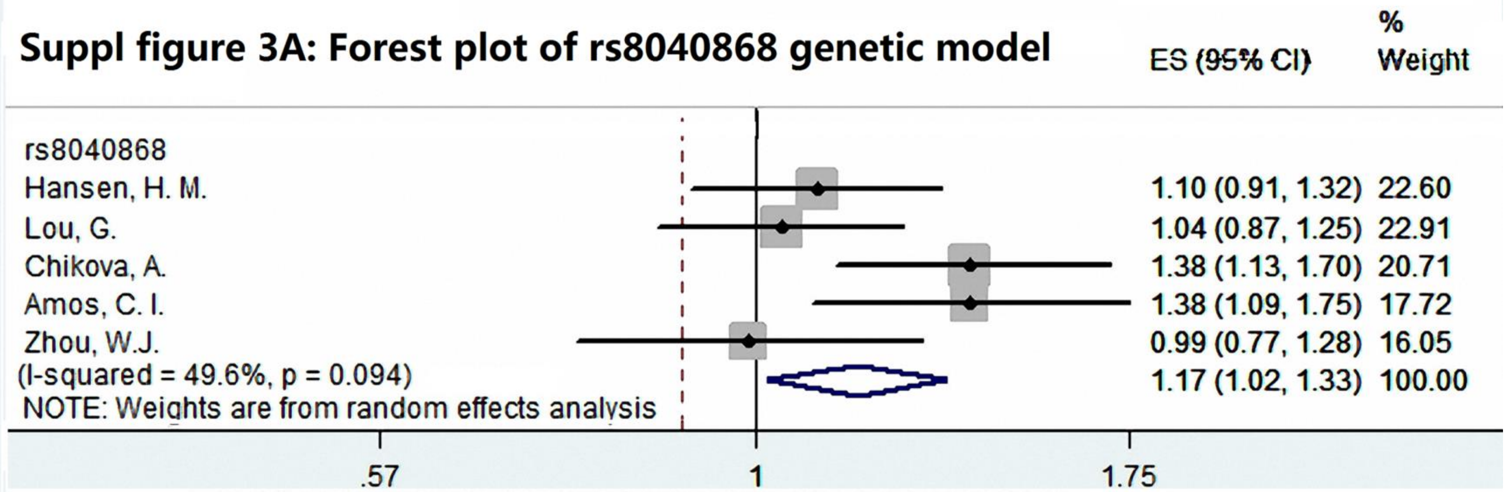

Suppl figure 3A: Forest plot of rs8040868 genetic model

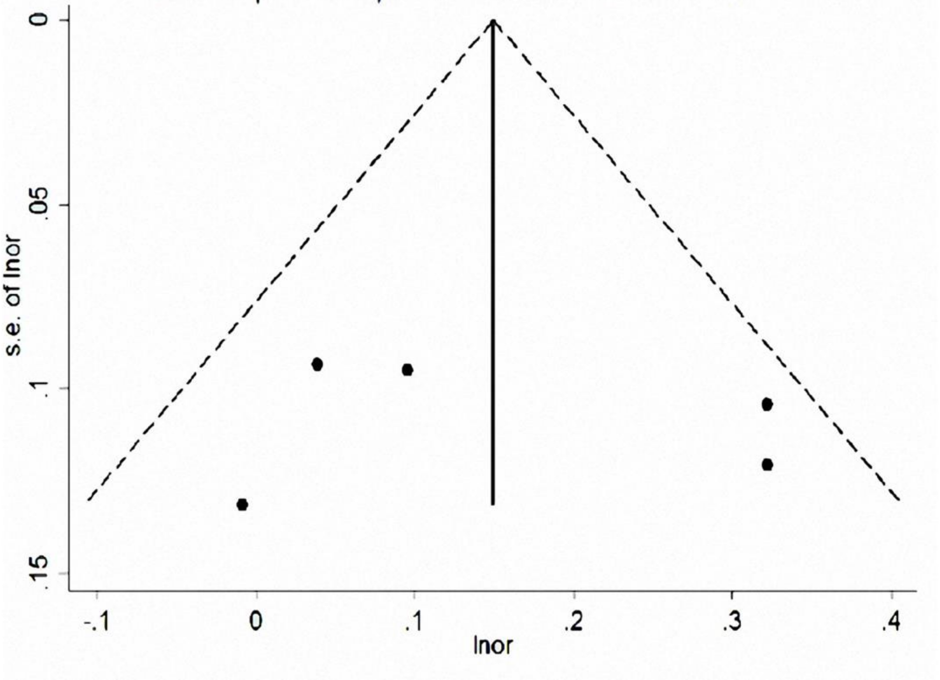

Suppl figure 3B: Rs8040868: Funnel plot with pseudo 95% confidence limits

Figure legends:

Supplementary figure 1 Sensitivity analysis for allele C analysis of rs938682 and genotype CC of rs938682. Tseng's and Broderick's research might be a potential source of heterogeneity for the allele C and CC genotype of rs938682 respectively.

Supplementary figure 2 Galbraith plot for allele C analysis and genotype CC for rs938682. The plot showed no statistically significant heterogeneity and no researches exceeded the heterogeneity limits.

Supplementary figure 3 Forest plot (3a) and funnel plot (3b) for rs8040868. Allele T was associated with increased risk of lung cancer with moderate heterogeneity (OR with 95%CI: 1.17(1.02-1.33), I^2=49.6%). No significant publication bias exists (p=0.783).
